# Experimental hut evaluation of a novel long-lasting non-pyrethroid durable wall lining for control of pyrethroid-resistant *Anopheles gambiae* and *Anopheles funestus* in Tanzania

**DOI:** 10.1186/s12936-017-1710-6

**Published:** 2017-02-17

**Authors:** Robert Malima, Basiliana Emidi, Louisa A. Messenger, Richard M. Oxborough, Bernard Batengana, Wema Sudi, Sophie Weston, George Mtove, Joseph P. Mugasa, Franklin W. Mosha, Mark W. Rowland, William Kisinza

**Affiliations:** 1 National Institute for Medical Research, Amani Research Centre, Muheza, Tanzania; 2 Department of Entomology and Parasitology, Kilimanjaro Christian Medical University College (KCMUCo) of Tumaini University, Moshi, Kilimanjaro Tanzania; 3 Department of Disease Control, London School of Hygiene and Tropical Medicine (LSHTM), London, UK

**Keywords:** Insecticide-treated wall lining, Long-lasting insecticidal nets, Malaria control, Experimental huts, Pyrethroid resistance

## Abstract

**Background:**

A novel, insecticide-treated, durable wall lining (ITWL), which mimics indoor residual spraying (IRS), has been developed to provide prolonged vector control when fixed to the inner walls of houses. PermaNet^®^ ITWL is a polypropylene material containing non-pyrethroids (abamectin and fenpyroximate) which migrate gradually to the surface.

**Methods:**

An experimental hut trial was conducted in an area of pyrethroid-resistant *Anopheles gambiae* s.l. and *Anopheles funestus* s.s. to compare the efficacy of non-pyrethroid ITWL, long-lasting insecticidal nets (LLIN) (Interceptor^®^), pyrethroid ITWL (ZeroVector^®^), and non-pyrethroid ITWL + LLIN.

**Results:**

The non-pyrethroid ITWL produced relatively low levels of mortality, between 40–50% for *An. funestus* and *An. gambiae*, across all treatments. Against *An. funestus*, the non-pyrethroid ITWL when used without LLIN produced 47% mortality but this level of mortality was not significantly different to that of the LLIN alone (29%, P = 0.306) or ITWL + LLIN (35%, P = 0.385). Mortality levels for *An. gambiae* were similar to *An. funestus* with non-pyrethroid ITWL, producing 43% mortality compared with 26% for the LLIN. Exiting rates from ITWL huts were similar to the control and highest when the LLIN was present. An attempt to restrict mosquito access by covering the eave gap with ITWL (one eave open *vs* four open) had no effect on numbers entering. The LLIN provided personal protection when added to the ITWL with only 30% blood-fed compared with 69 and 56% (P = 0.001) for ITWL alone. Cone bioassays on ITWL with 30 min exposure after the trial produced mortality of >90% using field *An. gambiae.*

**Conclusions:**

Despite high mortality in bioassays, the hut trial produced only limited mortality which was attributed to pyrethroid resistance against the pyrethroid ITWL and low efficacy in the non-pyrethroid ITWL. Hut ceilings were left uncovered and may have served as a potential untreated refuge. By analogy to IRS campaigns, which also do not routinely treat ceilings, high community coverage with ITWL may still reduce malaria transmission. Restriction of eave gaps by 75% proved an inadequate barrier to mosquito entry. The findings represent the first 2 months after installation and do not necessarily predict long-term efficacy.

## Background

Most malaria-endemic countries have adopted policies to promote universal distribution of long-lasting insecticidal nets (LLINs) free of charge across all age groups, and an estimated 49% of the population in sub-Saharan Africa had access to at least one LLIN in their household in 2013 [1]. However, resistance to pyrethroid insecticides used in all LLINs is now widespread across vector populations and may reduce the level of community protection [2, 3]. Another challenge is maintaining effective year-round protection as during the hot dry seasons when transmission may still occur, some individuals are deterred from sleeping under nets [4]. The development of large holes through wear and tear of net fabrics during normal household use may compromise their protective efficacy despite LLINs retaining insecticidal potency for three years [5]. In areas of hyper-endemic malaria transmission, even when universal coverage (UC) of LLINs is achieved and nets are in good condition, malaria prevalence can remain relatively high unless additional control tools are implemented [6].

Indoor residual spraying (IRS) is a proven vector control method that has been used since the Second World War and was the central feature of the Global Malaria Eradication Campaign between 1955 and 1969, which successfully eliminated malaria from several countries and significantly reduced disease incidence in others [7, 8]. In 2005 the US President’s Malaria Initiative (PMI) revived IRS in sub-Saharan Africa by funding an initial $1.2 billion programme in 15 countries [9]. IRS coverage in sub-Saharan Africa increased substantially from <2% of the at-risk population protected in 2005 to 11%, or 78 million people, by 2010 [1]. A major challenge facing IRS programmes is how to sustain such gains in the face of operational problems, such as vector resistance to insecticides, lack of affordable alternative insecticides and limited resources for recurrent annual campaigns. In addition to the labour costs associated with spraying, high levels of pyrethroid resistance among mosquito populations have necessitated the use of more expensive non-pyrethroid formulations [10]. The commodity cost of PMI-supported IRS campaigns with the organophosphate Actellic CS 300 (pirimiphos methyl CS) has been estimated to be more than four times the expense of using the pyrethroid Icon CS 10 (lambdacyhalothrin CS) to cover the same area [11].

A new product has been developed which mimics the effect of IRS but is designed to control insecticide-resistant mosquitoes for a minimum of three years. Insecticide-treated, durable wall lining (ITWL) is a material that can be fixed to the inner walls and ceilings of houses. The principle is the same as IRS, to kill mosquitoes that land on the ITWL either before or after blood-feeding. If the coverage of ITWL is high enough, the population density and longevity of mosquitoes in the area becomes substantially reduced, together with malaria transmission. There is also the possibility that ITWL could be used to block entry of mosquitoes if the material is extended from floor to ceiling, therefore covering eave spaces. While ITWL, like IRS, could be used to reduce transmission by itself, it is more likely to be an adjunct to LLINs, with the LLIN providing additional personal protection through the barrier and excito-repellent effect. ZeroVector^®^ is a first-generation ITWL containing deltamethrin incorporated into high-density polyethylene shade cloth, which has been evaluated in several countries in sub-Saharan Africa and Asia and consistently received high levels of household acceptability and provided prolonged insecticidal activity of greater than 12 months [12, 13]. With pyrethroid resistance now widespread throughout sub-Saharan Africa, attention has switched to development of a new generation of non-pyrethroid ITWL.

Initial experimental trials of ITWL + LLINs were conducted using plastic sheeting that had been spray treated with a non-pyrethroid (organophosphate) insecticide [14, 15]. These studies produced differing results in Côte d’Ivoire and Burkina Faso, which were attributed to variation in phenotypic resistance to organophosphates and pyrethroids among the respective vector populations [14, 16]. A newer factory-produced product (PermaNet^®^ Lining) has been developed that consists of thin non-woven sheets of cloth made from high-density polypropylene containing a non-pyrethroid insecticide mixture of abamectin (avermectin) and fenpyroximate (pyrazole) which are slowly released together and migrate to the surface of the fibre; neither insecticide has been used in malaria control before. Abamectin is a macrocyclic lactone that acts through chloride channel activation and was discovered in 1981 [17]. Contact bioassays have shown that abamectin is efficacious against house flies [18], cockroaches [19] and fire ants [20] in terms of mortality, and there is evidence for oviposition suppression in blowflies [21]. There are limited data for use against mosquitoes, but ivermectin (also an avermectin) is highly effective in terms of both mortality and oviposition suppression as a cattle parasiticide [22]. Abamectin is widely used in mixtures for control of crop and ornamental pests associated with greenhouse and nursery operators, e.g., abamectin + trifosine (fungicide) is used to control the two-spotted spider mite [23]. Fenpyroximate is a pyrazole in the mitochondrial complex 1 electron transport inhibitors (METI) group of insecticides, which disrupt insect respiration and are in widespread use globally. METI acaricides are extensively used to control *Tetranychus* spp (spider mites) [24]. To date, there are no published data demonstrating efficacy of these proprietary insecticides against mosquitoes.

Despite the promise of ITWL, the only existing data to support the efficacy of this new product are small-scale, unpublished studies conducted by the manufacturer. In response to the increasing problem of insecticide resistance, PMI has funded a large-scale, cluster-randomized controlled trial (CRT) in Muheza, Tanzania to investigate whether ITWL combined with UC of LLINs provides added protection against malaria compared with LLINs alone [25]. To aid interpretation of the CRT results, an experimental hut trial of the ITWL with or without LLIN was conducted in Muheza against wild free-flying populations of *Anopheles funestus* sensu stricto (s.s.) and *Anopheles gambiae* sensu lato (s.l.).

## Methods

### Insecticide treatments

The following insecticide treatments were tested in the experimental hut trial:

1. Untreated negative control (all eaves open);
2. Interceptor^®^ LLIN (alphacypermethrin 200 mg/m^2^, BASF, Germany) (all eaves open);
3. Non-pyrethroid ITWL (PermaNet^®^ Lining; abamectin 0.25%, fenpyroximate 1%, Vestergaard Frandsen, Switzerland) (full coverage on walls) (all eaves open);
4. Non-pyrethroid ITWL + Interceptor^®^ LLIN (all eaves open);
5. Non-pyrethroid ITWL (partially blocked eaves, only one of the four eaves left open);
6. Pyrethroid ITWL (ZeroVector^®^; deltamethrin 4.4 g/kg, Vestergaard Frandsen, Switzerland) (full coverage on walls) (all eaves open).

### Insecticide safety

With any new vector control product, it is essential that rigorous mammalian and environmental tests are undertaken to determine whether it is safe to use at the proposed dosages. Both active ingredients have low mammalian toxicity and good safety profiles [25]. The proprietary combination formula in the non-pyrethroid ITWL has passed an initial environmental examination (IEE) conducted by an independent regulatory agency; hazard quotients for continuous habitation in a residence with non-pyrethroid ITWL were far below the acceptable threshold [25].

### Experimental hut trial

An experimental hut trial was conducted at the National Institute for Medical Research (NIMR) field station at Zeneti village (5°13′S latitude, 38°39′E longitude and 193 m altitude) where *An. gambiae* s.l. and *An. funestus* s.s. are the major malaria vector species. Insecticide susceptibility tests using World Health Organization (WHO) diagnostic dosages showed that *An. gambiae* s.l. were fully susceptible to pyrethroids (permethrin, deltamethrin) in 2009–2010 [26]. By 2011 there were signs of pyrethroid resistance, with mortality of 75% for permethrin and deltamethrin [27]. In 2014 mortality of *An. gambiae* s.l. collected from experimental huts was 74% when exposed to deltamethrin, 51% for lambdacyhalothrin and 81% for permethrin [28]. Also in 2014, *An. funestus* s.s. were found to be resistant to deltamethrin and alphacypermethrin with mortality rates of 75 and 60%, respectively [28]. There is currently no resistance to carbamates or organophosphates.

**Fig. 1:**
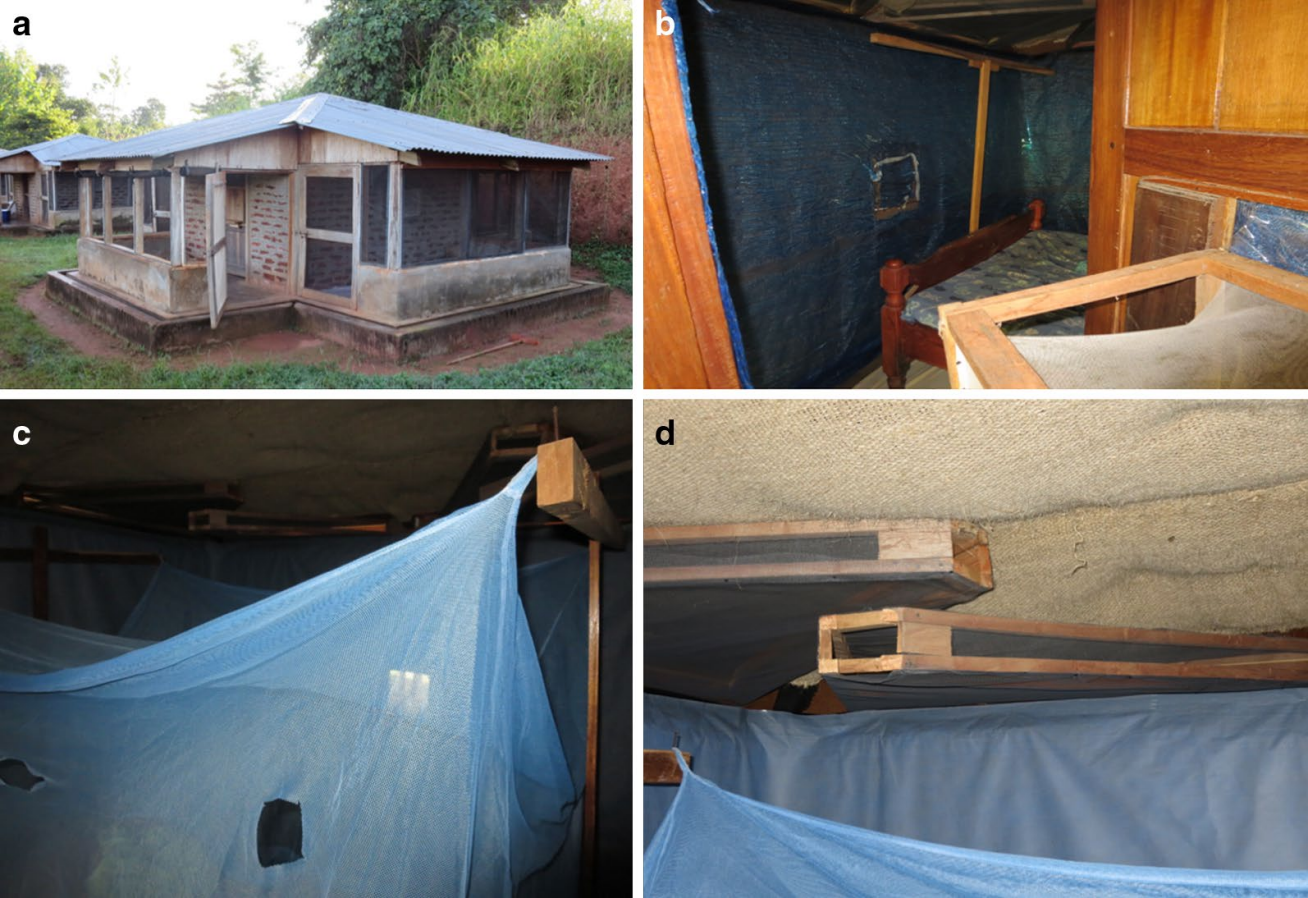
**a** East African experimental huts in Zeneti village, Muheza District, northeast Tanzania. **b** Pyrethroid ITWL (ZeroVector^®^). **c** Non-pyrethroid ITWL (PermaNet^®^ Lining) + LLIN (Interceptor^®^) with eaves partially blocked. **d** Eave baffles and hessian sack cloth ceiling

Experimental huts were constructed to a design described by the WHO [29] and based on the original veranda-hut developed in Tanzania in the 1960s [30, 31] (Fig. 1). In the modern design the eave gap is reduced to 5 cm, the ceiling board is lined with hessian sack cloth, similar to thatch, and the concrete floor surrounded by a water-filled moat [31]. In this trial the veranda traps were not used and the verandas left unscreened. This allowed mosquitoes to freely enter huts through all four eave spaces so the impact of partial eave blocking (only one eave left open) with ITWL could be assessed for some treatments. Inwardly directed eave baffles were installed to prevent egress of mosquitoes that had entered the hut; the only route of exiting was through the window traps [32, 33] (Fig. 1). LLINs were deliberately holed with six 4 × 4-cm holes to simulate wear and tear [29].

Six experimental huts were used in total. An adult volunteer slept in each hut nightly from 20:30–06:00. All were provided with chemoprophylaxis and instructed on use. The six volunteers were rotated between huts on successive nights to reduce any bias due to differences in individual attractiveness to mosquitoes. ITWL treatments were attached to wall boards using Velcro and rotated between huts after every seventh night (six trial nights and one non-trial night for cleaning and aeration with no treatment) for a total duration of 63 nights (49 trial nights). Mosquito collections were conducted using mouth-aspirators between 06:30–08:00 each morning by trained field assistants. White sheets were laid on the concrete floor in the room to ensure dead mosquitoes were more easily visible. Dead and live mosquitoes were collected inside the hut and from the two window traps (not from the verandas, as they were left unscreened). Live mosquitoes were transferred to 150-ml paper cups and provided with 10% glucose solution for scoring delayed mortality after 24, 48, 72 h at the NIMR laboratory. Gonotrophic status was recorded as unfed, blood-fed, semi-gravid, or gravid. All members of the *An. gambiae* species complex and *An. funestus* species group identified by morphological characteristics were assumed to be *An. gambiae* s.l. and *An. funestus* s.s. based on recent PCR identification [27].

The entomological impact of each treatment was expressed relative to the untreated control in terms of the following:

1. Induced mortality: percentage of dead mosquitoes in the treated hut at the time of collection and after a 72 h holding period relative to the control hut;
2. Deterrence: percentage reduction in the number of mosquitoes caught in the treated hut relative to the number caught in the control hut;
3. Induced exiting (repellency) due to any potential irritant effect of treatment expressed as percentage of mosquitoes collected from the veranda traps of treated huts relative to percentage caught in the veranda trap of the control hut;
4. Inhibition of blood-feeding: reduction in blood-feeding rate relative to the control. This was calculated using the following model: 100 (Bf_u_ –Bf_t_)/Bf_u_. where Bf_u_ is the proportion of blood-fed mosquitoes in the untreated control hut and Bf_t_ is the proportion of blood-fed mosquitoes in the huts with insecticide treatments.

### Supplementary bioassays

#### WHO cone and cylinder tests

To evaluate the efficacy of non-pyrethroid ITWL, standard WHO cone and cylinder bioassays were performed, based on the WHO protocol for IRS monitoring [29], using insectary-reared, pyrethroid-susceptible *An. gambiae* Kisumu and pyrethroid-resistant *An. gambiae* Muleba-Kis strains, and F1 generation of wild *An. gambiae* s.l. collected from five villages (Kibaoni-Mlingano, Kicheba, Mamboleo-Lusanga, Mkanyageni, Muungano) participating in the CRT. Bioassays were conducted with an exposure of 30 min to new pieces of non-pyrethroid ITWL from multiple batch productions and mortality recorded after 24, 48 and 72 h.

#### Irritability tests

To characterize any excito-repellent properties of the non-pyrethroid ITWL, 50 non-blood-fed, 2- to 5-day old, insectary-reared, pyrethroid-susceptible *An. gambiae* Kisumu or F1 generation of wild *An. gambiae* s.l. collected from Mkanyageni were individually introduced into plastic cones, containing either a piece of untreated netting, new non-pyrethroid ITWL or Interceptor^®^ LLIN, allowed to settle for 60 s, and time elapsed between the first landing and the next take-off of the mosquito was recorded, up to 360 s.

#### Effective exposure time

To determine the minimum exposure time required to kill 100% of mosquitoes exposed to the non-pyrethroid ITWL, WHO cylinder tests were conducted using progeny of wild *An. gambiae* s.l. which were exposed to new pieces of non-pyrethroid ITWL for 1.87, 3.75, 7.5, 15, 30 and 60 min and mortality recorded after 24, 48 and 72 h. Five replicates of ten mosquitoes were tested for each exposure time. Parental mosquitoes were collected from two villages participating in the CRT (Kicheba and Mamboleo-Lusanga).

### Data analysis

Data were entered into an Excel database and transferred to Stata 11 for processing and analysis (Stata Corp LP, College Station, TX, USA). For the experimental hut trial, the principal aim was to compare the efficacy of the different treatments relative to the untreated control. The outcomes of interest were proportion of mosquitoes blood-fed, dead (i.e., total number of mosquitoes dead by morning plus delayed mortality after holding for a total of 72 h) and exiting on successive nights. Logistic regression for grouped data was used to estimate the outcomes, comparing results for treatments and untreated control, adjusting for clustering by day and for variation between individual sleepers and huts. Negative binomial regression was used to analyse numbers entering the huts (% deterrence). Bioassay data were summarized using proportions and means and binomial confidence intervals, where applicable.

## Results

**Fig. 2:**
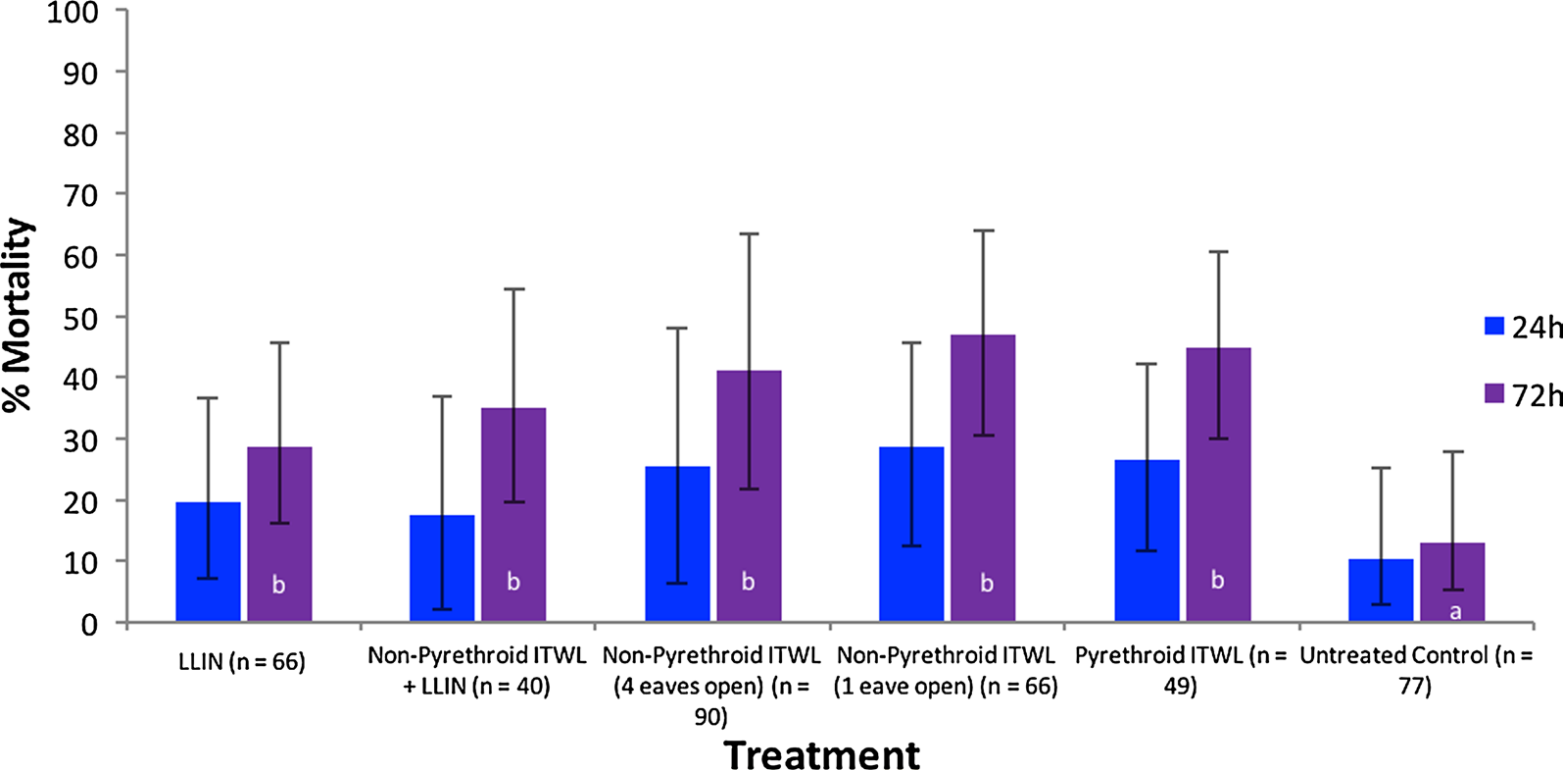
Percentage mortality of pyrethroid-resistant *Anopheles funestus* s.s. in the experimental hut trial. Percentage mortality was recorded 24 and 72 h after morning collections from huts. If the superscript for a time period (24 or 72 h) is the same, there was no significant difference between treatments (P > 0.05)

**Fig. 3:**
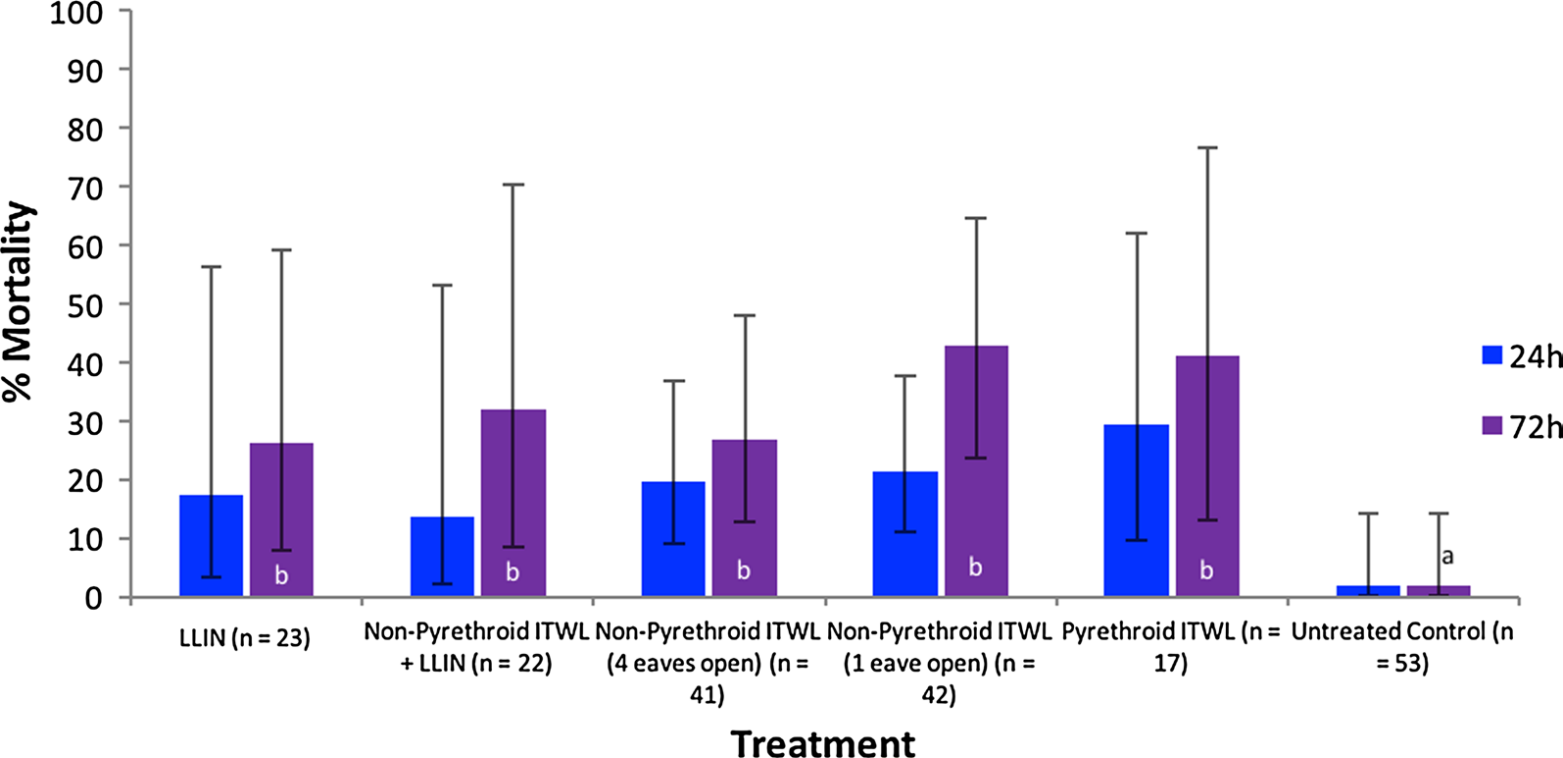
Percentage mortality of pyrethroid-resistant *Anopheles gambiae* s.l. in the experimental hut trial. Percentage mortality was recorded 24 and 72 h after morning collections from huts. If the superscript for a time period (24 or 72 h) is the same, there was no significant difference between treatments (P > 0.05)

Mortality results (24 and 72 h) are presented in Fig. 2 for *An. funestus* s.s. and Fig. 3 for *An. gambiae* s.l. Results showing deterrence, insecticide-induced exiting and reduction in blood-feeding are presented in Table 1 (*An. funestus* s.s.) and Table 2 (*An. gambiae* s.l.).

**Table 1:** Effect of insecticide-induced deterrence, exiting and blood-feeding of *Anopheles funestus* s.s. in the experimental hut trial

Insecticide treatment	N	Deterrence	% Exited by morning	% Insecticide-induced exiting	% Blood-feeding	% Blood-feeding inhibition
LLIN (Interceptor^®^)	66^ab^	14	89^b^ (75–96)	33	24^a^ (12–43)	15
Non-pyrethroid ITWL + LLIN	40^b^	48	75^bc^ (53–89)	20	30^ac^ (15–50)	0
Non-pyrethroid ITWL (4 eaves open)	90^ab^	0	34^d^ (19–55)	0	69^b^ (46–85)	0
Non-pyrethroid ITWL (1 eave open)	66^ab^	14	58^ac^ (41–73)	0	56^bd^ (38–73)	0
Pyrethroid ITWL (ZeroVector^®^)	49^ab^	36	88^b^ (74–95)	32	45^cd^ (28–63)	0
Untreated control	77^a^	NA	60^acd^ (49–70)	NA	29^a^ (17–44)	NA

If the superscript in a column is the same there was no significant difference between treatments (P > 0.05)

**Table 2:** Effect of insecticide-induced deterrence, exiting and blood-feeding of *Anopheles gambiae* s.l. in the experimental hut trial

Insecticide treatment	N	Deterrence	% Exited by morning	% Insecticide-induced exiting	% Blood-feeding	% Blood-feeding inhibition
LLIN (Interceptor^®^)	23^b^	57	91^b^ (65–98)	48	44^a^ (22–68)	0
Non-pyrethroid ITWL + LLIN	22^b^	59	86^bc^ (64–96)	45	36^ab^ (21–56)	0
Non-pyrethroid ITWL (4 eaves open)	41^ab^	23	39^a^ (14–71)	0	73^b^ (40–92)	0
Non-pyrethroid ITWL (1 eave open)	42^a^	21	60^ac^ (44–73)	21	55^b^ (40–69)	0
Pyrethroid ITWL (ZeroVector^®^)	17^b^	68	65^ac^ (38–85)	27	29^ab^ (12–57)	18
Untreated control	53^a^	NA	47^a^ (31–64)	NA	36^a^ (21–55)	NA

If the superscript in a column is the same there was no significant difference between treatments (P > 0.05)

### *Anopheles funestus* s.s

Mortality 72 h after mosquito collections was relatively low across all treatments (Fig. 2). Interceptor^®^ is a WHOPES-recommended pyrethroid LLIN but produced only 29% mortality. The non-pyrethroid ITWL when used without LLIN produced 47% mortality (eaves partially blocked) but this was not significantly different to that of the LLIN (29%, P = 0.306), non-pyrethroid ITWL + LLIN (35%, P = 0.385), or pyrethroid ITWL (45%, P = 0.306). Mortality of non-pyrethroid ITWL increased between 24 and 72 h after mosquito collections and accounted for 38% of total mortality. However, there was also an unexpected 32% delayed mortality for the pyrethroid LLIN. Levels of mortality to the non-pyrethroid ITWL were consistently low across the 7 week trial, demonstrating no significant decline in bioefficacy (38, 44 and 35% 72 h mortality of *An. funesus* s.s. after one to two, two to four and >4 weeks, respectively, for non-pyrethroid ITWL with open eaves).

The trial was conducted at the end of the rainy season and numbers of *An. funestus* s.s. collected were relatively few (n = 77 in the control). There was no significant difference in the numbers that entered huts with non-pyrethroid ITWL when eaves were partially blocked or open (P = 0.9562) (Table 1). The only treatment which produced any measurable deterrence effect was the non-pyrethroid ITWL + LLIN which reduced entry by 48% (P = 0.044). There was also a significant increase in the proportion of *An. funestus* s.s. that had exited into window traps by morning for the pyrethroid LLIN (P = 0.001) and pyrethroid ITWL (P = 0.001) treatments compared with the untreated hut. The non-pyrethroid ITWL produced no significant increase in exiting (P = 0.082). The LLIN provided added personal protection over the non-pyrethroid ITWL with only 24% blood-fed compared with 69% (P = 0.001) and 56% (P = 0.001) for the two ITWL treatments (eaves open and partially closed). The blood-feeding rate in the untreated hut was surprisingly low, and significantly less than in the huts with ITWL.

### Anopheles gambiae sensu lato

The numbers of *An. gambiae* s.l. collected over the duration of the trial were few (n = 53 in the control), meaning that the sample size was generally too small to assess differences between treatments (except where the effect was particularly large). Mortality levels 72 h after mosquito collections were uniformly low across all treatments, with the non-pyrethroid ITWL producing 43% mortality (eaves partially closed) compared with just 26% for the LLIN (Fig. 3). The mortality levels were similar to those for *An.* funestus s.s. and there were no significant differences between treatments. There was a degree of delayed mortality between 24 and 72 h, as observed previously with *An. funestus* s.s. The LLIN (57%, P = 0.007), non-pyrethroid ITWL + LLIN (59%, P = 0.002) and pyrethroid ITWL (68%, P = 0.002) all resulted in significant deterrence of *An. gambiae* s.l. entering indoors (Table 2). However, the non-pyrethroid ITWL treatment alone did not produce such an effect (P = 0.093 for eaves partially closed, and P = 0.393 for eaves open). As observed with *An. funestus* s.s., there was no significant difference in the numbers that entered when eaves were partially blocked or open (P = 0.956). The pyrethroid LLIN (48%, P = 0.002) and non-pyrethroid ITWL + LLIN (45%, P = 0.004) also produced a significant increase in the proportion of *An. gambiae* s.l. that had exited into window traps by morning. The non-pyrethroid ITWL did not increase exiting compared to the untreated hut. The LLIN (44% blood-fed) demonstrated significantly greater blood-feeding inhibition than the non-pyrethroid ITWL (73% for eaves open, and 55% for eaves partially closed).

### Bioassay results

#### WHO cone and cylinder tests

**Fig. 4:**
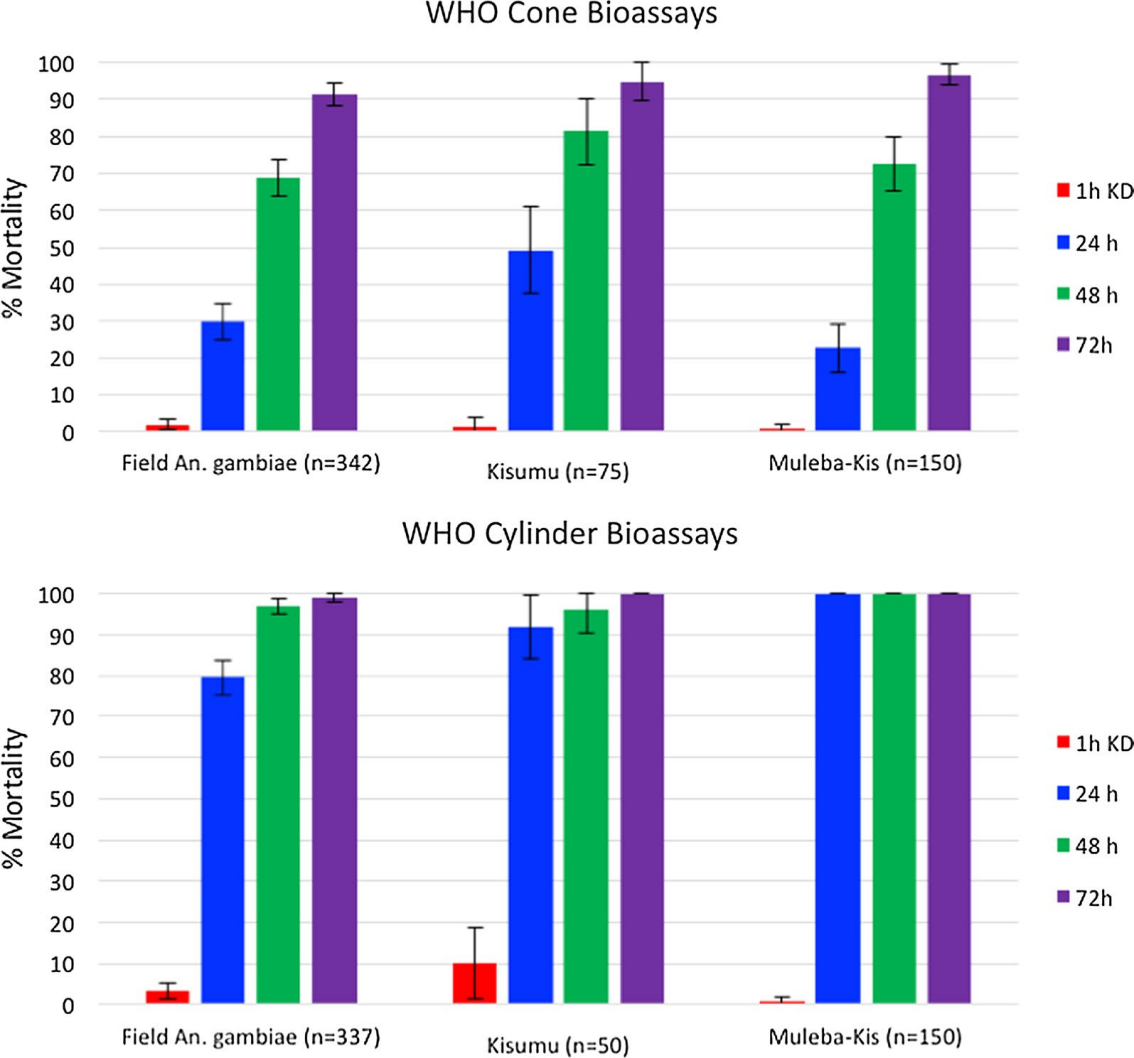
Percentage mortality of pyrethroid-susceptible *Anopheles gambiae* Kisumu, pyrethroid-resistant *Anopheles gambiae* Muleba-Kis and F1 offspring of field-collected *Anopheles gambiae* s.l. in WHO cone and cylinder bioassays on non-pyrethroid ITWL

WHO cone and cylinder bioassays with non-pyrethroid ITWL samples from five batch productions, conducted with an exposure time of 30 min, showed no significant difference in mortality between ITWL pieces (95 and 99% mortality after 72 h in cone and cylinder assays, respectively). When replicates were pooled, a direct comparison of cone *versus* cylinder bioassays demonstrated that overall immediate (after 24 h) and delayed (after 72 h) mortality was significantly lower in cone than cylinder bioassays (23–49 vs 80–100% mortality at 24 h, and 91–97 vs 99–100% mortality at 72 h for cones vs cylinders, respectively; P = 0.001 for all) for all mosquito strains tested (Fig. 4).

#### Irritability tests

**Fig. 5:**
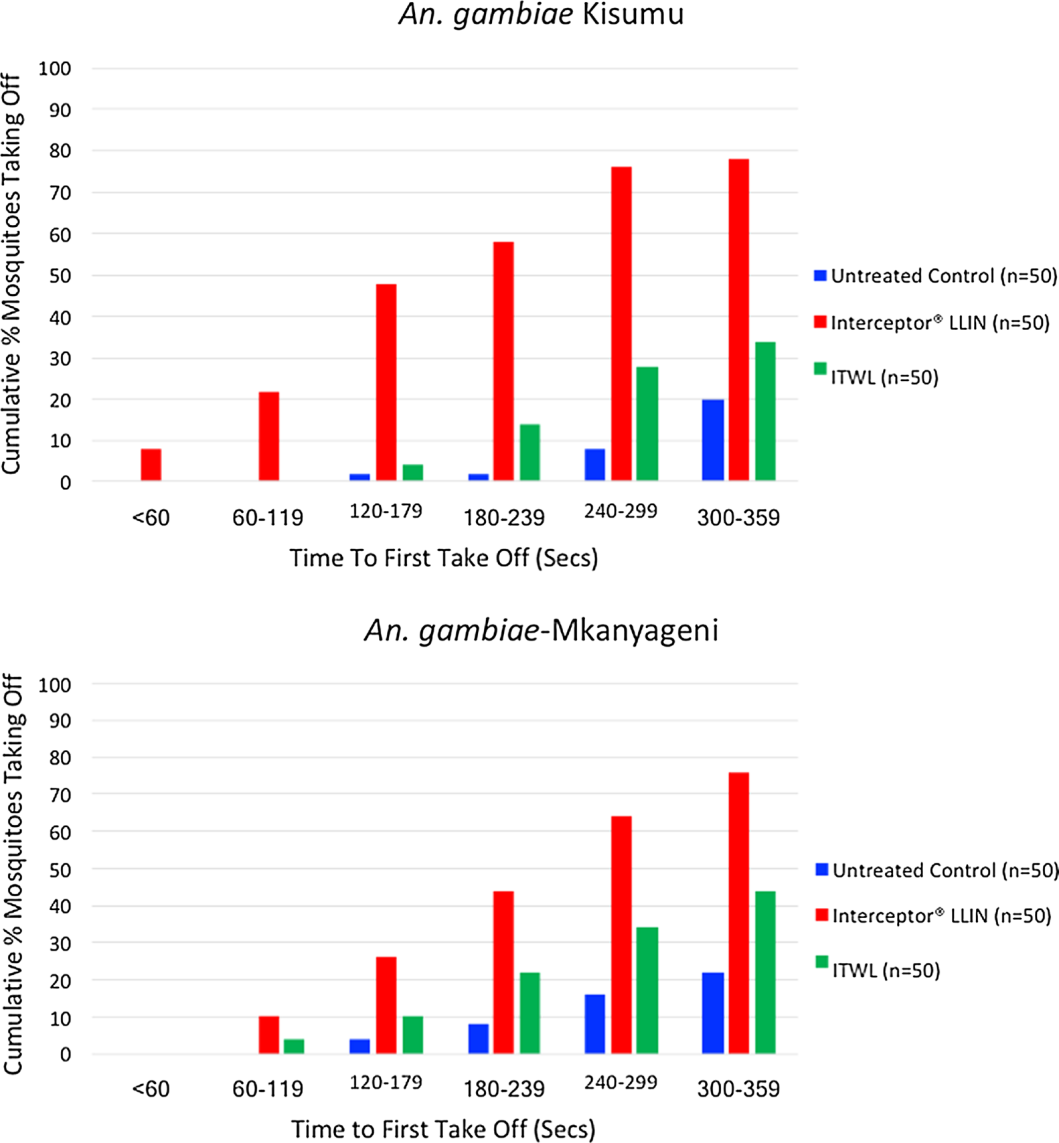
Cumulative percentage of pyrethroid-susceptible *Anopheles gambiae* Kisumu, and F1 offspring of field-collected *Anopheles gambaie* s.l. taking off over time, following exposure to untreated netting, Interceptor^®^ LLIN or non-pyrethroid ITWL

Time to first take-off in response to non-pyrethroid ITWL exposure was measured among individual *An. gambiae* Kisumu and F1 wild *An. gambiae* s.l. mosquitoes in comparison to untreated netting and a pyrethroid LLIN (Interceptor^®^ LLIN). By 6 min of exposure to non-pyrethroid ITWL, 34 and 44% of *An. gambiae* Kisumu and wild mosquitoes had taken flight, respectively, compared to 78 and 76% in response to Interceptor^®^ LLIN and 20 and 22% in untreated controls (Fig. 5). No mosquito irritability or excito-repellency was detected in response to non-pyrethroid ITWL exposure, as evidenced by no significant difference in numbers of mosquitoes taking flight, over the 6 min, in response to the non-pyrethroid ITWL compared to the untreated control (P = 0.232 for *An. gambiae* Kisumu and P = 0.425 for wild *An. gambiae* s.l.).

#### Effective exposure time

**Fig. 6:**
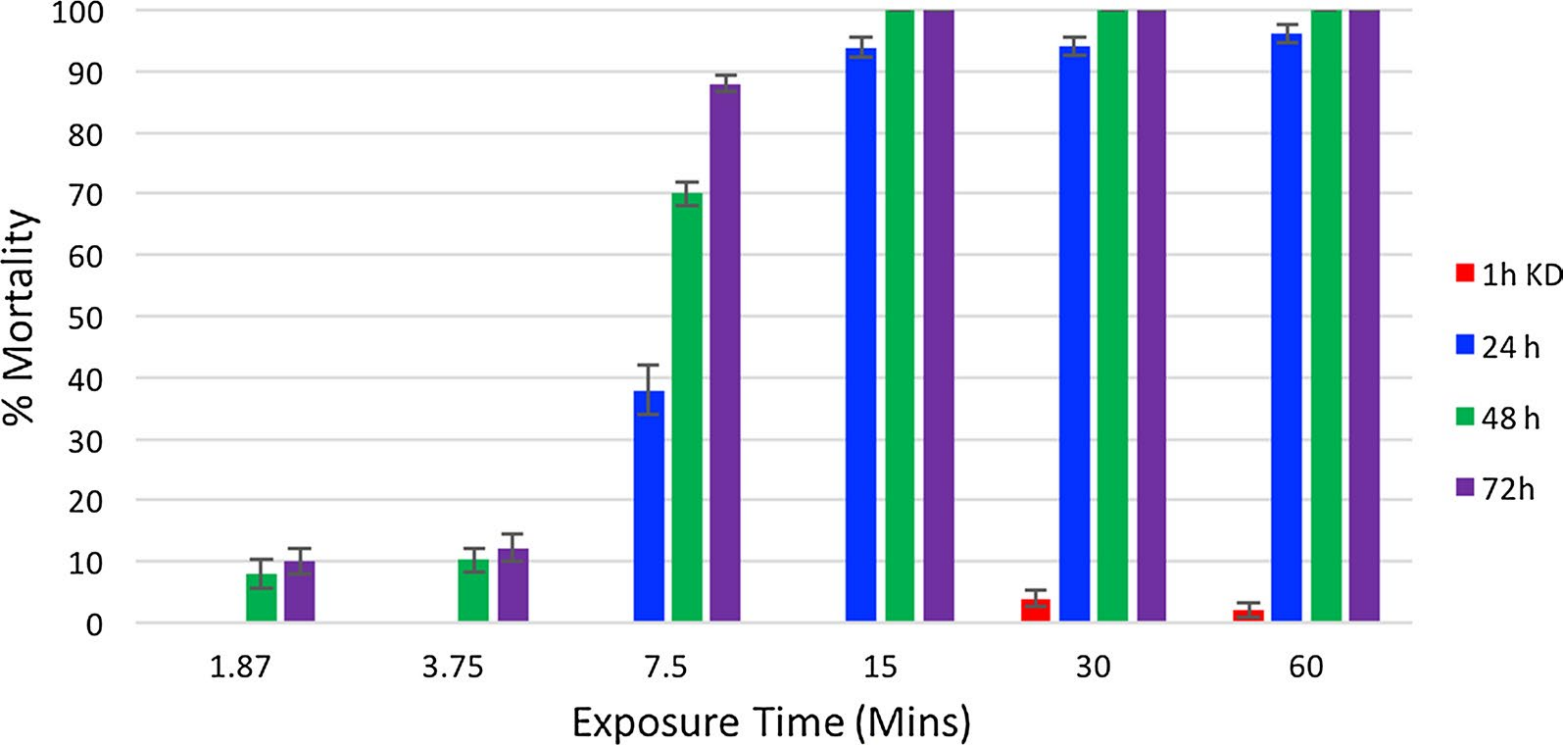
Percentage mortality of F1 offspring of field-collected *Anopheles gambiae* s.l. in WHO cylinder bioassays, following different exposures times to non-pyrethroid ITWL

An exposure time of 7.5 and 15 min to new non-pyrethroid ITWL in WHO cylinder tests killed 88 and 100% of F1 wild *An. gambiae* s.l. mosquitoes, respectively, after a 72 h holding period (Fig. 6). Exposure time of 15 min was sufficient to kill 100% of mosquitoes within 48 h.

## Discussion

At present there are no recognized WHO standards for ITWL products. Before progressing to community trials, candidate IRS and LLIN products are evaluated in Phase II experimental huts against an existing gold standard positive control and comparative performance assessed [29, 34]. In this trial the positive control was the WHOPES-recommended pyrethroid LLIN, which produced equivalent levels of mortality to the non-pyrethroid ITWL. However, due to pyrethroid resistance in both of the major vector species, the level of mortality for the LLIN was lower than previous reports from the same site [28]. In 2006 when vector species were fully susceptible to pyrethroids, Interceptor^®^ LLIN (alphacypermethrin) killed 92% of *An. gambiae* s.l. when unwashed [35], while Olyset^®^ LLIN (permethrin) produced 61% mortality for *An. gambiae* s.l. and 72% for *An. funestus* s.s. [36]. The mortality rate with Interceptor^®^ LLIN in this trial was considerably lower at just 26% for *An. gambiae* s.l. and 29% for *An. funestus* s.s. and was due to the significant increase in resistance across local vector populations [26]. In this study the non-pyrethroid ITWL also elicited relatively low levels of mortality, between 40 and 50% for *An. funestus* s.s. and *An. gambiae* s.l. The level of mortality in free-flying mosquitoes remained consistent over the 7 weeks of the trial.

WHO cone and cylinder bioassays exposing laboratory-reared susceptible and wild resistant *An. gambiae* s.l. to new pieces of non-pyrethroid ITWL for 30 min produced 90–100% mortality, demonstrating the inherent toxicity of the ITWL insecticides, with no evidence for cross-resistance to pyrethroids. In both assays, mosquito mortality remained low at 24 h, reaching the highest levels after 72 h. While new formulations would ideally produce more immediate mortality, a similar phenomenon has been reported for chlorfenapyr, a pyrrole insecticide which has demonstrated promise as an IRS and net treatment [37, 38]; historically some organochlorines used successfully as IRS (e.g., dieldrin) were also characterized by delayed mortality.

To help interpret the low levels of mortality observed in the main trial, WHO cylinder assays with incremental exposure times were performed to assess the duration of contact time required to kill the field strain (F1 progeny) of *An. gambiae* s.l. Only 7.5–15 min of contact with the non-pyrethroid ITWL induced 80–100% mortality. In all assays, levels of mosquito mortality were comparable between different rolls of ITWL, excluding differences in manufacturer batch production as a potential confounder in the main trial.

With the demonstration of high mortality in the bioassays, the lower levels in the main trial may be attributable to wild, free-flying mosquitoes spending less time in contact with treated wall surfaces. Shorter resting times are expected when insecticides induce a degree of repellence or irritancy. To further characterize mosquito behaviour in relation to ITWL exposure, time to first flight was measured for *An. gambiae* Kisumu and F1 wild *An. gambiae* s.l. in comparison to untreated netting and a pyrethroid LLIN (Interceptor^®^ LLIN). No significant irritability was observed on exposure to the non-pyrethroid ITWL, unlike the pyrethroid LLIN which is known to have excito-repellent properties. These results were supported by the low exiting rates of both *An. funestus* s.s. and *An. gambiae* s.l. in non-pyrethroid ITWL huts compared to pyrethroid treatments (Interceptor^®^ LLIN and ZeroVector^®^ ITWL) in the main trial.

In the main trial, only treatments containing pyrethroid interventions significantly reduced vector entry. By comparison, the low levels of deterrence in the non-pyrethroid ITWL huts and the supporting irritability bioassays indicate that the ITWL was not influencing mosquito entry. One possible explanation for the low mortality, exiting and deterrence in the non-pyrethroid ITWL huts, is that vectors were instead resting on the hessian sack cloth ceilings, which remained uncovered throughout the trial, and were not contacting the walls for sufficient time to obtain a lethal insecticide dose. During the trial no data were documented on the location of mosquitoes within the room. While few studies have characterized exactly where African malaria mosquitoes reside within experimental huts, a recent trial of pirimiphos-methyl IRS on wooden panelled walls performed at the same study site also reported unexpectedly low levels of mortality and mosquitoes were noted to be resting on sack cloth-lined ceiling (M. Rowland, unpublished data). Earlier experimental hut trials of insecticide-treated wall lining materials have demonstrated that efficacy is strongly correlated with intervention surface area, with increasing coverage affording higher rates of vector mortality, deterrence and blood-feeding inhibition [39, 40]. If in this trial mosquitoes secured refuge on the ceiling over the non-woven polyethylene wall lining material, this could partly explain the low mortality in the huts. During IRS campaigns in Tanzania, ceilings are not usually sprayed, being too high and inaccessible to spray men, and yet such campaigns can have had a major effect on *An. gambiae* population density and malaria transmission rates [6].

It has been suggested that ITWL may also impact malaria transmission by functioning as a method of housing improvement, if used to cover eave gaps, preventing mosquito ingress [25]. However, in the present trial, there was no significant differences in vector entry in huts with partially blocked or open eaves. It is possible that host-seeking mosquitoes are able to compensate for a partial restriction of ‘entry points’ if host odour is concentrated from those that remain. ITWL material is not currently designed to act an eave seal or withstand strong winds and house improvements would better look to other means of restricting access.

## Conclusions

PermaNet^®^ ITWL is a new malaria vector control strategy, containing two non-pyrethroid insecticides, which is designed to function as a long-lasting IRS, when fixed to the inner walls and ceiling of houses. An experimental hut trial was performed in Muheza, Tanzania, to evaluate performance during 2 months after installation in comparison with a WHOPES-recommended LLIN (Interceptor^®^) and pyrethroid ITWL (ZeroVector^®^). The level of mosquito mortality was lower than expected: in the pyrethroid-treated LLIN and pyrethroid ITWL this was explained by insecticide resistance; in the non-pyrethroid ITWL this was attributed to low efficacy; since the ceiling was uncovered some mosquitoes may have secured refuge on this untreated surface. A series of novel, supplementary bioassays characterized the toxicity and mode of action of the non-pyrethroid ITWL product, and its effect on mosquito behaviour. The findings represent the first 2 months after installation and do not necessarily predict longer-term residual efficacy of non-pyrethroid ITWL.

## Abbreviations

AI: active ingredient
CRT: cluster-randomized controlled trial
CS: capsule suspension
IEE: initial environmental examination
IRS: indoor residual spraying
ITWL: insecticide-treated wall lining
KCMC: Kilimanjaro Christian Medical College
LLIN: long-lasting insecticidal net
LSHTM: London School of Hygiene and Tropical Medicine
METI: mitochondrial complex 1 electron transport inhibitors
MRCC: Medical research coordinating committee
NA: not applicable
NIMR: National Institute of Medical Research
PMI: US President’s Malaria Initiative
UC: Universal coverage
WHO: World Health Organization
WHOPES: WHO Pesticide Evaluation Scheme
